# Motoring Notes

**Published:** 1909-07-31

**Authors:** 


### Motoring Notes.

### The Single-cylinder Car.

Having frequently in these columns urged the manifold advantages of the small single-cylinder car for the medical motorist's use as compared with the larger type with many cylinders it gives me pleasure to quote the opinion of Mr. Douglas Fawcett, the well-known motorist. Writing in *The Motor* he says: "A word to the small car owner who is apt to shrink from travelling abroad. There is no occasion to tour in a large, or even in a medium-sized car; touring in couples in small two seaters is great fun. Resort to powerfully-engined cars is entirely unnecessary. You can cross France in an eight h.p. car from Havre or Boulogne and reach the Alps easily in three or four days with far more appreciation of your surroundings than you would have if you rushed along in a dust-raising behemoth which is devouring a set of tyres. I would not exchange my small two-seater for anything which runs on the road, and assuredly I would not be bothered by four cylinders or, indeed, complications of any kind. Time was when folk would write of the 'noisy propensities' of one-cylinder cars, and the expression lingers yet, I regret to note, in the pages of the *Motor Manual.* But the best one-cylinder cars of to-day are quieter than were four-cylinders two years ago. In fact my own small De Dion (B.N. 1909) is one of the quietest cars I have come across this year. The folk at Chamounix frequently express surprise, comparing it very favourably with its larger sisters here, and one garage proprietor actually came out and congratulated me on the possession of my neat and silent little 'four.' Anxious as ever to further the cult of the one-cylinder car—in my opinion, the best all-round motor vehicle yet constructed for couples who tour far afield—I will give in brief the reasons which induce me to recommend it:—(1) Complete reliability and simplicity combined with comfort. (2) Ease of the 'garage' work. No morning overhaul—a turn of the handle and you are off. Any greasing, etc., which may be necessary from time to time can be done in odd moments. (3) Obvious lightness on tyres a small two-seater with 760 mm. by 90 mm. covers, and thick raised tread, is practically immune from punctures, while the general tyre wear is of little account. Let me add that I have driven five De Dion one-cylinder cars and have never had to buy a new cover. The re-treading of two back covers has accounted for all my extra outlay. (4) Petrol consumption is low, and in view of the ease of maintaining efficiency in the case of one-cylinder (firing point admitting of perfect adjustment as you run and carburation being perfected by free use of air lever) is easily kept so. (5) The small car is particularly satisfactory on very long steep climbs, four-cylinder cars often overheating in the Alps. It does not, I find, ever suffer from such overheating. (6) The best small cars wear very well. The one-cylinder engine, *e.g.* does not require to be 'taken down' after its first or second season; a hideous necessity and a costly one to boot. (7) Pace ample. I may say that in a test made by me between Langres and Gray in a recent run from Paris to Switzerland, 59 kilom., or say 37 miles were covered in one and a quarter hours, giving for a vehicle with engine of only 100 mm. by 120 mm. the average of $29\frac{3}{5}$ miles per hour. The same car ascends to Chamounix on the second speed (where necessary) at 16 or 17 miles per hour. In view of the enormous field of pleasure touring open to such a car, I want nothing better. Complications as favoured by the owners of small four-cylinder cars I have no use for." It is a source of satisfaction that the type of car which has been consistently advocated in these columns as the most suitable for a medical man's use, especially the doctor who does not keep a chauffeur, should be so strongly recommended by a motorist of Mr. Douglas Fawcett's reputation. Mr. Fawcett does not of course allude to the single-cylinder car as suitable for the medical profession, but it is obvious that a car which possesses so many advantages for continental touring and the very arduous work of Alpine climbing, in which it is superior to the larger multi-cylinder type, must of necessity be the vehicle most suitable for the hard every-day work of the country doctor.

### Correct Proportion of Gas and Air.

In these days of automatic carburettors the control of the mixture is on many cars taken out of the hands of the driver. This may seem an advantage to the novice, but every experienced driver and especially old motor-cyclists know well that automatic carburation can never be quite satisfactory under all running conditions. On cars which are not fitted with this latest "improvement" and in which the mixture is controlled by the driver it is advisable to experiment with the air opening so as to obtain the best mixture which is suited to the density of the atmosphere—such experimenting, of course, is necessary, as the proportion of air will vary with climatic conditions, though the carburettor is not so sensitive with a larger engine and with a spray carburettor as to necessitate alteration in the proportion of air in a slight change of such conditions. Clearly the air openings on many carburettors might well be larger; otherwise additional air valves would not be in such demand or add so much to power and petrol economy. "Viator."

